# Prophylactic bilateral internal iliac artery balloon occlusion with immediate sheath removal for placenta accreta spectrum

**DOI:** 10.1186/s42155-026-00649-z

**Published:** 2026-01-17

**Authors:** Sooyeon Joy Kim, Natalie Layden, Scott Fleming, Hasan İlksen Hasan, Amin Bahabri, Sarah Louise Rylance, Gurjeet Singh Dulku

**Affiliations:** 1 Medical Imaging and Interventional Radiology Department, Fiona Stanley and Fremantle Hospitals Group, South Metropolitan Health Service, Murdoch, WA 6150 Australia; 2 Obstetrics and Gynaecology Department, Fiona Stanley Hospital, South Metropolitan Health Service, Murdoch, WA 6150 Australia

**Keywords:** Interventional radiology, Placenta accreta spectrum, Prophylactic balloon occlusion, Postpartum haemorrhage, Uterine artery embolisation

## Abstract

**Background:**

Consistent with global trends, the incidence of placenta accreta spectrum (PAS) is increasing in Australia. Prophylactic internal iliac arterial balloon occlusion (PIIABO) is an endovascular intervention utilised to assist haemorrhage control during caesarean delivery in women with PAS, offering a potentially uterus-preserving alternative to hysterectomy. However, existing outcomes remain heterogeneous. This study aimed to evaluate the endovascular safety of PIIABO with immediate sheath removal in the management of PAS, with a secondary assessment of haemostatic and procedural outcomes.

**Materials and methods:**

A 10-year retrospective, single-centre cohort study of all patients with suspected PAS who underwent PIIABO was conducted with data obtained from electronic medical records and Radiology Information System (RIS)/Picture Archiving and Communication System (PACS).

**Results:**

Fifteen patients underwent PIIABO. The mean maternal age was 34.1 years, with a mean gravidity of 4.2 and a parity of 2.3; all had prior caesarean delivery and 93% had concurrent major placenta praevia (*n* = 14). Mean gestational age at delivery was 34.9 weeks. Diagnosis was established by MRI (*n* = 11, 87.5% concordance) and ultrasound (*n* = 4, 50% concordance). Twelve patients underwent hysterectomy, confirming 1 accreta, 3 increta, and 8 percreta; 3 patients preserved uterus, with intraoperative evidence of percreta (*n* = 2) or normal placentation (*n* = 1). Mean estimated blood loss was 2273 mL, and 11 patients received blood transfusions, including four who required ≥ 4 units of packed red blood cells. Mean balloon inflation time was 129.9 min, sheath dwell time 265.5 min, and operating theatre time 265.7 min. Mean dose-area product was 55.03 Gy.cm^2^ with a mean fluoroscopy time of 10.7 min. Radiation exposure decreased by approximately 90% over the study period with increasing institutional experience. No endovascular complications or reinterventions occurred, and all mothers and neonates were discharged without long-term morbidity.

**Conclusion:**

PIIABO with immediate sheath removal demonstrated favourable procedural outcomes and a low complication rate in patients with PAS, supporting its safe implementation within a multidisciplinary care pathway.

## Background

Placenta accreta spectrum (PAS) is a potentially life-threatening obstetric condition characterised by abnormal placental adherence and invasion, which is classified into three subtypes on the basis of the depth of placental invasion: placenta accreta, when villi adhere to the myometrium; placenta increta, when villi invade the myometrium; and placenta percreta, with full thickness invasion beyond the myometrium and into the serosa, with possible involvement of adjacent pelvic organs [1]. This pathological invasion prevents normal placental separation at delivery and is associated with a high risk of massive obstetric haemorrhage. The incidence of PAS has increased globally over recent decades, with Australian data reporting an incidence of 47.4 per 100,000 pregnancies, largely attributed to rising maternal age, caesarean delivery rates, and the prevalence of concurrent placenta praevia [2, 3].

Planned caesarean hysterectomy has been regarded as the definitive management strategy for PAS owing to the risk of uncontrolled peripartum haemorrhage; however, this approach results in permanent loss of fertility and adverse psychological outcomes, prompting interest in conservative and uterus-preserving alternatives [4–6]. Prophylactic balloon occlusion is a minimally invasive endovascular procedure increasingly employed as an adjunct in the conservative management of PAS. This involves preoperative endovascular placement and inflation of balloons in the infrarenal aorta, common iliac, or internal iliac arteries to reduce pelvic blood flow following delivery of the foetus. Proposed benefits include reduced intraoperative blood loss, decreased transfusion requirements, and potentially lower rates of maternal morbidity and mortality, especially in patients with significant placental invasion [7].

Despite its increasing utilisation, evidence regarding the efficacy and safety of prophylactic balloon occlusion remains inconsistent, attributable to variation in study design, procedural technique, and institutional resources, which collectively limit the development of robust evidence-based recommendations [7]. Reported endovascular complication rates vary widely, ranging from minor complications such as claudication to major thromboembolic events leading to distal thrombosis requiring further intervention, especially when arterial sheaths were left in situ postoperatively in anticipation of delayed endovascular intervention [7–9]. Across Australian centres, balloon placement sites have varied between the common and internal iliac arteries, reflecting differing institutional management protocol and the rationale that more proximal occlusion may provide broader suppression of pelvic arterial inflow by addressing collateral pathways [10–14].

At the authors’ institution, prophylactic internal iliac artery balloon occlusion (PIIABO) is the preferred balloon occlusion technique performed in a hybrid operating theatre with immediate removal of all endovascular instrumentation at the completion of the operation. This approach was adopted on the premise that maintaining lower limb perfusion by leaving external iliac arteries patent and minimising sheath dwell time may reduce vascular complications while preserving the option for rapid selective uterine artery embolisation (UAE) should haemorrhage persist.

This study aimed to evaluate safety, haemostatic profile, and the procedural outcomes of PIIABO with immediate sheath removal in the management of PAS at a single Australian tertiary centre.

## Materials and methods

### Study design and patient enrolment

This study received approval from the institutional human research ethics committee (Governance, Evidence, Knowledge, Outcomes ID 56126) and was conducted in accordance with Strengthening the Reporting of Observational Studies in Epidemiology (STROBE) guideline. A retrospective, single-centre cohort study was conducted at the sole tertiary referral centre in Western Australia offering PIIABO as part of multidisciplinary management for PAS.

All patients with antenatally suspected PAS identified on imaging who subsequently underwent PIIABO from the inception of the hospital in October 2014 to March 2025 were eligible for inclusion. Patients who underwent PIIABO for indications other than suspected PAS and patients with known bleeding disorders were excluded. No formal sample size calculation was performed as this study included all consecutive patients meeting eligibility criteria, representing the complete institutional experience with PIIABO.

### Institutional setup and PIIABO technique

**Fig. 1:**
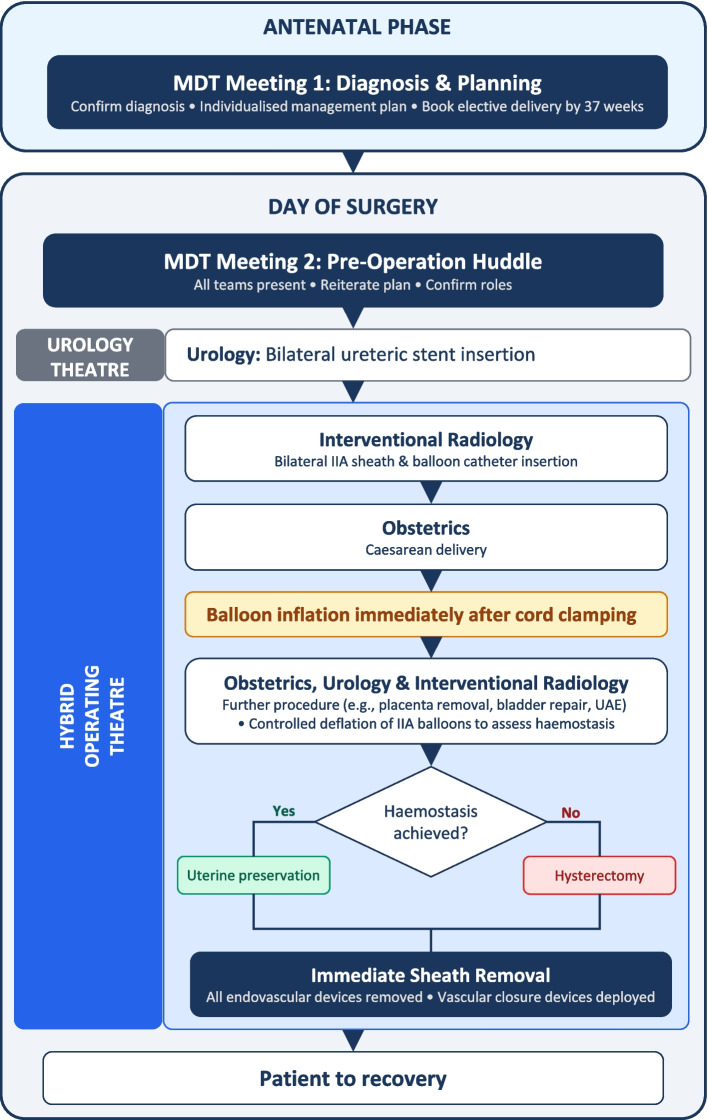
Flow diagram detailing the multidisciplinary approach to management of placenta accreta spectrum

Patients were managed through a multidisciplinary team (MDT) which involves interventional radiologists, obstetricians, maternal-foetal medicine specialists, urologists, anaesthesiologists, neonatologists, perioperative services, and midwifery consultants. Each case was discussed at least twice during the antenatal period to establish the diagnosis and refine an individualised perioperative management plan as outlined in Fig. 1. Caesarean hysterectomy with PIIABO was the default approach; however, uterine preservation was considered when intraoperative haemostasis was achieved. Cell salvage was utilised alongside allogenic blood products where appropriate. Written informed consent was obtained prior to the procedure.

**Fig. 2:**
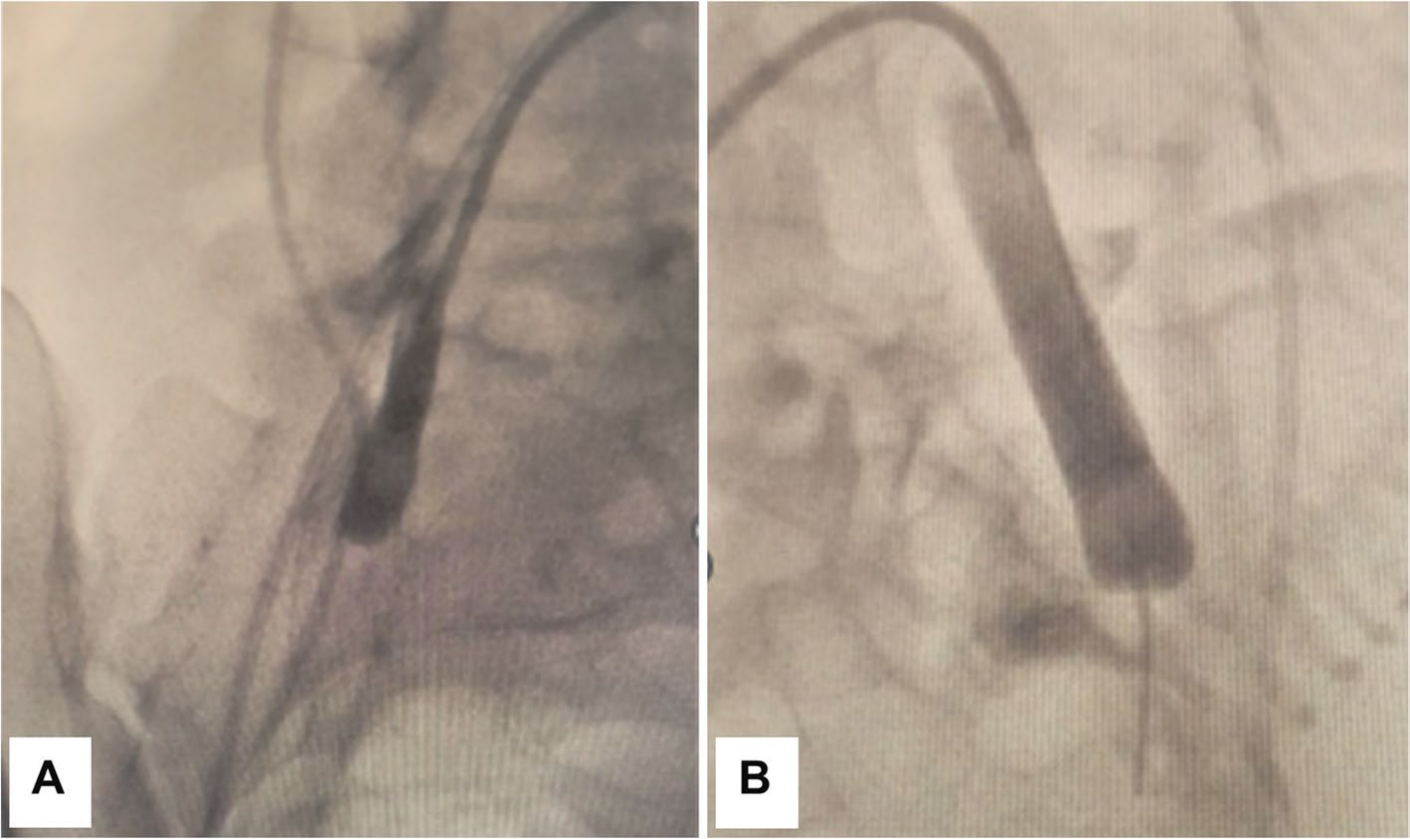
Test inflation of Fogarty balloons in the right (**A**) and the left (**B**) internal iliac artery. No contrast flow is demonstrated distal to the inflated balloons

Bilateral common femoral access was obtained under ultrasound guidance with 5-Fr sheaths, which were exchanged for 6-Fr, 45 cm Destination sheaths (Pinnacle Destination, Terumo Interventional Systems, Somerset, NJ, USA). 5.5-Fr Fogarty balloon catheters (Edwards Lifesciences, USA) were positioned at the proximal internal iliac arteries and test-inflated to determine the volume required to occlude antegrade flow. The endovascular sheaths and balloon catheters were secured to the skin with sutures and adhesive dressings. Continuous saline infusion was connected to each sheath and balloon catheter to maintain patency, and distal perfusion was monitored via pulse oximetry on each great toe. Balloons were inflated immediately following umbilical cord clamping and intermittently deflated to assess haemostasis. If haemorrhage persisted, UAE were performed in the same setting. Vessel patency was confirmed fluoroscopically or with digital subtraction angiography (DSA) as required. All endovascular devices were removed at the completion of the operation, and vascular closure devices were deployed. Access site and lower limb neurovascular status were monitored regularly for evidence of endovascular complications (Fig. 2).

### Clinical outcomes and statistical analysis

Patients were identified through medical records and RIS/PACS using keyword-based searches and followed for 90 days postoperatively. To minimise information bias, data were independently extracted by two investigators (SJK and NL) using a standardised approach, with discrepancies resolved by senior author consensus.

The primary outcome was endovascular safety of PIIABO with immediate sheath removal. Endovascular complications were defined as access-site haematoma, gluteal claudication, thrombosis, distal embolism, pseudoaneurysm, or arterial dissection. Secondary outcomes were haemostatic and perioperative measures which included estimated blood loss (EBL), blood products transfused within 24 h of surgery, procedural parameters (hybrid theatre time, sheath dwell time, and balloon inflation time), and radiation metrics (dose-area product [DAP] and fluoroscopy time). EBL was determined intraoperatively by clinical estimation based on suction canister volume. Surgical complications deviating from the expected perioperative course were graded using the Clavien–Dindo classification [15]; planned urological procedures for known bladder involvement in placenta percreta were not classified as complications.

Outcomes were stratified by PAS subtype. Histopathologic confirmation was used for patients undergoing hysterectomy; in conservatively managed cases, subtype was based on intraoperative findings, supplemented by antenatal imaging.

Fisher’s exact and Mann–Whitney *U* tests were used for two-group comparisons, and the Kruskal–Wallis test for comparisons involving more than two groups. Temporal trends in radiation exposure were assessed by comparing cases performed before and after January 2023, representing a transition from early to established institutional experience. Statistical significance was set at *P* < 0.05.

An internal propensity-matched analysis was not feasible due to the absence of a contemporaneous non-PIIABO cohort, as PIIABO has been incorporated into standard practice at the study institution since its inception. For descriptive context, outcomes were compared with a published cohort of high-risk obstetric patients treated at a tertiary women's hospital within the same metropolitan region [16]. Although the study population was not exclusive to PAS, a defined subgroup of patients with PAS who underwent elective caesarean hysterectomy without endovascular intervention was identified. Individual-level data were unavailable, precluding formal comparative analysis; therefore, this comparison should be interpreted with caution.

## Results

### Patient demographics

**Fig. 3:**
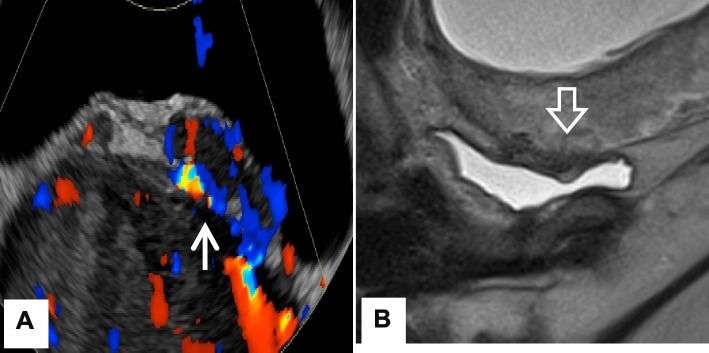
Antenatally suspected placenta percreta with **A** transvaginal ultrasound demonstrating myometrial thinning with several tortuous vessels (←) between the uterus and bladder, and **B** T2-weighted coronal MRI demonstrating thinning of myometrium and superior bladder wall irregularity(⇦) without definite bladder wall invasion

Fifteen patients with antenatally suspected PAS underwent PIIABO during the study period; no patients met exclusion criteria. Patient demographics are elaborated on Table 1. Placenta percreta was the most common subtype (*n* = 10, 67%), followed by increta (*n* = 3, 20%) and accreta (*n* = 1, 7%). One patient with sonographic features suggestive of placenta accreta and equivocal MRI findings was found to have normal placentation at surgery (Fig. 3). Twelve patients underwent planned caesarean hysterectomy, while uterine preservation was achieved in three patients (20%), including two with placenta percreta and one with normal placentation. Among patients in whom uterine preservation was achieved, information regarding desire for future pregnancy and subsequent fertility outcomes was unavailable. No endovascular complications were observed following PIIABO with immediate sheath removal.

**Table 1:** Patient demographics

	**Results (*n* = 15)**
Maternal age	34.1 (20–42)
Maternal age > 35 years	9 (60%)
Gravidity	4.2 (2–16)
Parity	2.3 (1–10)
Gestational age at delivery, weeks	34.9 (31.9–37.6)
Prior Caesarean section	
1	6 (40%)
2 or more	9 (60%)
PAS subtype	
Normal placentation	1 (7%)
Accreta	1 (7%)
Increta	3 (20%)
Percreta	10 (67%)
Concurrent placental previa	
Absent	1 (7%)
Major	14 (93%)
Uterus preservation	3 (20%)
Length of ICU admission, d	1.2 (0–7)
Length of postoperative hospitalisation, d	6.1 (3–15)

Data shown in mean (range) or *N* (%)

### Haemostatic outcomes

Haemostatic outcomes stratified by PAS subtype are presented in Table 2. The mean EBL was 2273 mL, with greater blood loss (> 2500 mL) observed in 4 patients (26.7%); however, no statistically significant difference was observed across PAS subtypes (*P* = 0.75). When contextualised against the external non-PIIABO cohort [16], estimated blood loss in our cohort was numerically lower (Table 3). Eleven patients (73%) received blood products (3 patients with increta and 8 patients with percreta), with mean PRBC of 2.7 units (range 0–7) and mean cell salvage transfusion volume of 598 mL (range 250–2000 mL). Four patients received ≥ 4 units of PRBC, the majority of whom had underlying percreta (*n* = 3, 75%). No patient received ongoing blood transfusions beyond 24 h.

**Table 2:** Haemostatic outcomes and procedural parameters according to placenta accreta subtype

		**Normal (*n* = 1)**	**Accreta (*n* = 1)**	**Increta (*n* = 3)**	**Percreta (*n* = 10)**	**Total (*n* = 15)**	***P*-value**
EBL, mL	Mean (range)	2000	1000	2000 (1000–3500)	2510 (800–7000)	2273.3 (800–7000)	0.747^†^
	Median [IQR]					2000 [1250–2750]	
PRBC, units	Mean (range)	0	0	1.7 (0–4)	3.1 (0–7)	2.7 (0–7)	0.48^‡^
	Median [IQR]					3 [0.5–4]	
Cell salvage, mL	Mean (range)	0	0	302.7 (250–658)	484.6 (250–2000)	598 (250–2000)	0.86^‡^
	Median [IQR]					419 [295–525]	
Theatre time, mins	Mean (range)	192	223	321.7 (290–355)	260.5 (180–349)	265.7 (180–355)	0.1419^†^
	Median [IQR]					277 [215.5–299.5]	
Sheath dwell time, mins	Mean (range)	150	204	215 (187–229)	298.4 (157–986)	265.5 (150–986)	0.4411^†^
	Median [IQR]					204 [176.5–252]	
Balloon inflation time, mins	Mean (range)	97	136	142 (125–161)	129 (54–235)	129.9 (54–235)	0.7878^†^
	Median [IQR]					130 [94.5–151]	
DAP, Gy.cm^2^	Mean (range)	41.5	19.6	68.1 (48.1–85.2)	56 (3.3–298.2)	55.03 (3.3–298.2)	0.354^†^
	Median [IQR]					41.5 [12.8–60]	
FT, mins	Mean (range)	9.1	15	11.5 (5.4–20.1)	10.2 (4.4–30.6)	10.7 (4.4–30.6)	0.7181^†^
	Median [IQR]					9.1 [6.2–10.5]	

*P*-values are based on comparisons of median values using non-parametric testing; mean (range) is provided for descriptive comparison

*EBL* estimated blood loss, *PRBC* packed red blood cells, *DAP* dose-area product, *FT* fluoroscopy time

^†^*P*-values are based on Kruskal–Wallis testing

^‡^*P*-values are based on Mann–Whitney *U* test

**Table 3:** Descriptive comparison with external non-PIIABO cohort*

	**PIIABO cohort**	**External non-PIIABO cohort**
Study design	Retrospective, single-centre study	Retrospective, single-centre study
Study population	Suspected PAS undergoing PIIABO	High-risk obstetric patients receiving intraoperative autologous transfusion
Sample size	15	11 participants with PAS out of total 21
PAS subgroup	1 without PAS, 1 accreta, 3 increta, 10 percreta	6 accreta, 1 increta, 4 percreta
Concurrent PP	1 without PP, 14 major PP	11 major PP
EBL, mean (range)	2273 mL (800–7000 mL)	3136 mL (500–8500 mL)
PRBC, mean (range)	2.7 units (0–7)	3.2 units (0–14)
Cell salvage, mean (range)	598 mL (250–2000 mL)	419 mL (80–1000 mL)

*PIIABO* prophylactic internal iliac artery balloon occlusion, *PAS* placenta accreta spectrum, *PP* placenta praevia, *EBL* estimated blood loss, *PRBC* packed red blood cells

*This comparison is presented for contextual purposes only. Individual-level data were unavailable, precluding propensity-matched or formal statistical analysis. Differences in study populations, time periods, and outcome definitions limit direct comparison. Findings should be interpreted with caution and do not support causal inference

### Procedural and radiation metrics

Two cases deviated from the standard institutional protocol. In one case, bilateral internal iliac artery sheaths were pre-emptively inserted the night prior to operation in anticipation of possible emergency delivery, resulting in a prolonged sheath dwell time of over 16 h. The patient ultimately proceeded with planned PIIABO and caesarean hysterectomy the following morning without complication related to the extended sheath dwell time. In a second case, bilateral UAE using gelatine sponge (Spongostan; Ferrosan Medical Devices A/S, Søborg, Denmark) was performed following PIIABO, based on the intraoperative assessment of haemostasis. Despite underlying placenta percreta, the patient did not require blood transfusion and uterine preservation was achieved. This case was associated with a higher radiation exposure (DAP of 298.23 Gy.cm^2^; fluoroscopy time of 30.6 min), consistent with inherently higher radiation demands of embolisation procedures.

Procedural parameters are summarised in Table 2. The mean theatre time was 265.7 min, mean sheath dwell time was 265.5 min, and mean balloon inflation time was 129.9 min. Mean DAP was 55.03 Gy.cm^2^ and the mean fluoroscopy time was 10.7 min (Table 4). The case involving concurrent UAE contributed to right-skewing of the DAP; excluding this case, the mean DAP decreased to 37.7 Gy.cm^2^. Temporal analysis revealed a marked reduction in radiation exposure with increasing institutional experience. Cases performed after January 2023, representing the latter four of the cohort, demonstrated approximately 90% lower mean DAP compared with earlier cases (5.0 Gy.cm^2^ vs. 50.7 Gy.cm^2^, *P* = 0.002), despite no significant difference in operation time or fluoroscopy time (Table 5). This reduction coincided with the progressive implementation of radiation minimisation strategies, including tight collimation, pulsed fluoroscopy, and selective use of DSA.

**Table 4:** Radiation metrics of PIIABO cases

Case no	PAS subtype	Procedure	Theatre time, min	DAP, Gy.cm^2^	FT, min	DSA runs, N
1	Percreta	PIIABO + C + UAE	215	298.23	30.6	5
2	Percreta	PIIABO + CH + Partial cystectomy	295	87	5.7	10
3	Percreta	PIIABO + C	180	23.45	10.7	0
4	Accreta	PIIABO + CSH	223	19.56	15	0
5	Percreta	PIIABO + CH + Partial cystectomy	277	36.42	8.4	2
6	Increta	PIIABO + CH + L salpingectomy	290	71.03	20	3
7	Increta	PIIABO + CH + L salpingectomy	355	48.07	9	0
8	Without PAS	PIIABO + C	192	41.52	9.1	0
9	Increta	PIIABO + CSH	320	85.19	5.4	6
10	Percreta	PIIABO + CH + Bilateral salpingectomy + Bladder repair	214	47.48	9.5	0
11	Percreta	PIIABO + CSH + Bilateral salpingectomy	216	47.69	9.9	0
12	Percreta	PIIABO + CSH + Bilateral salpingectomy	282	5.32	5.6	0
13	Percreta	PIIABO + CH + Bilateral salpingectomy + Bladder repair	304	5.25	10.4	0
14	Percreta	PIIABO + CSH + Bladder repair	349	6	6.8	0
15	Percreta	PIIABO + CSH + Bladder dissection	273	3.3	4.4	0

*C* caesarean delivery, *CH* caesarean hysterectomy, *CSH* caesarean and subtotal hysterectomy, *FT* fluoroscopy time, *DAP* dose-area product, *DSA* digital subtraction angiography

**Table 5:** Radiation metrics by institutional experience period

	**Before Jan 2023**^**†**^** (*n* = 10**^**‡**^**)**	**After Jan 2023 (*n* = 4)**	***P*-value**
Mean DAP, Gy.cm^2^	50.7 (19.6–87)	5 (3.3–6)	0.002*
Mean number of DSA runs, *N*	2.1 (0–10)	0	0.184
Mean FT, minutes	10.3 (5.4–15)	6.8 (4.4–10.4)	0.19
Mean theatre time, minutes	256 (180–355)	302 (273–349)	0.36

*DAP* dose-area product, *DSA* digital subtraction angiography, FT fluoroscopy time

^**†**^January 2023 was selected as the threshold representing the transition from early to established institutional experience, after which consistent implementation of radiation minimisation strategies was achieved

^**‡**^One case involving combined PIIABO and UAE was excluded due to substantially higher radiation exposure related to embolisation

*P-value < 0.05, considered significant

#### Complications

No endovascular complications, re-laparotomy, or further endovascular intervention occurred. Four patients experienced minor surgical complications with Clavien-Dindo grade II or lower, including urinary tract infection (*n* = 2), surgical wound infection (*n* = 1), and postoperative ileus (*n* = 1); all were managed conservatively without long-term sequelae. Six patients with known bladder involvement with planned urological procedures were not classified as complications. All mothers and neonates were discharged without long-term complications.

## Discussion

The recent meta-analysis encompassing 19 PIIABO, including three randomised control trials (RCTs) and two prospective studies, demonstrated a statistically significant reduction in pooled EBL by 342.1 mL (95% confidence interval [CI]: − 509.9 to − 174.2 mL) compared with control cohorts [7]. The reported complication rate varied widely (1.4–20.8%), with higher complication rates consistently observed in studies where arterial sheaths were left in situ for longer durations [7–9, 17–21]. The finding is biologically plausible given the prothrombotic state of pregnancy, compounded by endothelial injury, stasis, and foreign body presence; factors highlighted in neurointerventional literature where overnight femoral sheaths promoted clot formation [22]. Prompt device removal reduces endothelial trauma and minimises vascular foreign body exposure, thereby mitigating thrombotic risk without compromising the option for same-session embolisation if required.

Several alternative balloon occlusion techniques have been evaluated through meta-analyses and demonstrated more proximal approaches such as prophylactic abdominal aorta balloon occlusion (PAABO) and prophylactic common iliac artery balloon occlusion (PCIABO) resulted in reduced EBL [7, 23–26]. The recent meta-analysis demonstrated strong haemostatic outcomes of PBOAA against control (mean difference [MD]: − 926.42 mL, 95% confidence interval [CI]: − 1437.07 mL to − 415.77 mL, *P* = 0.004), and PIIABO (MD—411 mL, 95% CI: − 779.7 mL to − 41.5 mL, *P* < 0.001) [7]. However, these haemostatic advantages have not consistently translated into differences in transfusion volume, thrombotic complications, or hysterectomy rates [24].

Despite evidence supporting greater haemorrhage reduction with proximal balloon occlusion, a key advantage of PIIABO lies in its ability to facilitate rapid conversion to selective embolisation through pre-positioned arterial access, enabling faster embolisation than distal branch cannulation required for PAABO or PCIABO in the setting of acute haemorrhage, where time is of the essence [27]. Same-session UAE following prophylactic balloon occlusion has emerged as a combined strategy to further optimise haemorrhage control. A randomised controlled study and multiple retrospective studies demonstrated reduced intraoperative blood loss and lower hysterectomy rates with combined PIIABO and UAE compared with conventional caesarean hysterectomy [28–31]. UAE itself is well established as an effective rescue and adjunctive therapy, with a meta-analysis of 1142 women reporting haemostatic success rates exceeding 90%, significant reductions in blood loss, and shorter hospital stays compared with caesarean hysterectomy [32].

Overall, current evidence demonstrates that both proximal and distal arterial balloon occlusion techniques, with or without concurrent UAE, reduce perioperative blood loss over caesarean hysterectomy [33]. However, major international guidelines reflect ongoing uncertainty regarding the optimal endovascular management of PAS. A systematic review of 14 international guidelines by Bonanni et al. identified a lack of consensus and poor uptake on the routine use of balloon occlusion catheters, primarily due to limited high-quality evidence and variable access to interventional radiology [34]. In contrast, UAE is endorsed by numerous major guidelines including the Cardiovascular and Interventional Radiology Society of Europe (CIRSE), International Federation of Gynecology and Obstetrics (FIGO), French College of Gynaecologists and Obstetricians (CNGOF), and the American College of Obstetricians and Gynaecologists (ACOG) for refractory postpartum haemorrhage prior to considering hysterectomy, where appropriate expertise is available [35–38].

A notable secondary finding was the approximately 90% reduction in radiation exposure observed in cases performed after January 2023, corresponding to the latter third of our institutional experience. This improvement occurred without prolongation of operative or fluoroscopy time, indicating that radiation minimisation can be achieved without compromising procedural efficiency. The reduction was attributable to progressive implementation of dose-reduction strategies and decreased reliance on DSA. A case–control study comparing uterine artery embolisation road-mapping techniques demonstrated significantly lower radiation exposure with conventional road mapping compared with DSA, consistent with our institutional experience [39]. Centres implementing PIIABO programmes should implement early adoption of radiation minimisation strategies to lower radiation dose for women and foetus.

Several limitations warrant consideration. The retrospective design, small sample size, and single centre setting limit generalisability, and outcomes may not be replicable in centres with less experience in PAS management or without hybrid operating theatre facilities. The absence of a contemporaneous internal comparator precluded formal comparative analysis, and reliance on medical records may have resulted in incomplete, inaccurate, or inconsistently documented data. Estimated blood loss was determined by clinical estimation, which is subject to inter-observer variability. Nevertheless, as the sole tertiary centre in Western Australia offering PIIABO, this cohort likely represents most PAS cases managed with this technique within the state. Based on published data, approximately 15 PAS cases would be expected in Western Australia annually [3, 40]. Many women may therefore undergo hysterectomy without access to fertility-sparing alternatives, which remain limited outside specialised multidisciplinary centres.

## Conclusion

The present study demonstrates that PIIABO with immediate sheath removal is feasible and associated with favourable procedural outcomes and a low incidence of endovascular complications in the management of PAS. Importantly, the ability to rapidly transition to concurrent uterine artery embolisation in cases of refractory haemorrhage provides additional haemostatic flexibility and may facilitate uterine conservation in selected cases, supporting PIIABO as a versatile adjunct in PAS management. Larger prospective comparative studies are required to better define the optimal endovascular strategy for conservative management of PAS.

## Data Availability

The datasets used and/or analysed during the current study are available from the corresponding author on reasonable request.

## Abbreviations

DAP: Dose-area product
DSA: Digital subtraction angiography
EBL: Estimated blood loss
FT: Fluoroscopy time
MDT: Multidisciplinary team
PACS: Picture Archiving and Communication System
PAS: Placenta accreta spectrum
PAABO: Prophylactic abdominal aorta balloon occlusion
PCIABO: Prophylactic common iliac artery balloon occlusion
PIIABO: Prophylactic internal iliac artery balloon occlusion
PRBC: Packed red blood cells
RIS: Radiology Information System
STROBE: Strengthening the Reporting of Observational Studies in Epidemiology
UAE: Uterine artery embolisation
